# Changing Trends in Phthalate Exposures

**DOI:** 10.1289/ehp.1408629

**Published:** 2014-10-01

**Authors:** Paul J. Lioy, Chris Gennings, Russ Hauser, Holger M. Koch, Andreas Kortenkamp

**Affiliations:** 1 Department of Environmental and Occupational Medicine, Rutgers Biomedical and Health Sciences-Robert Wood Johnson Medical School, Rutgers Environmental and Occupational Health Sciences Institute, Piscataway, New Jersey, USA; 2 Department of Biostatistics, Virginia Commonwealth University, Richmond, Virginia, USA; 3 Department of Environmental Health, Harvard School of Public Health, Boston, Massachusetts, USA; 4 Institute for Prevention and Occupational Medicine of the German Social Accident Insurance Institute of the Ruhr-Universität Bochum (IPA), Bochum, Germany; 5 Institute for the Environment, Brunel University, Uxbridge, United Kingdom

Two articles in the March 2014 issue of *EHP* were of particular interest to exposure scientists and phthalate researchers. The news article by Nicole (2014) emphasized the importance of the vaginal epithelium as an exposure route for chemicals in personal care products and the general lack of research on this exposure route. However, the article did not mention adult toys as a possible source of exposure. Adult toys are made from a variety of plastics, including polyvinyl chloride, and they may contain phthalates, some of which are associated with developmental effects in males following *in utero* exposure in both animal and epidemiological studies. One report has suggested that certain combinations of vehicle (personal lubricant) and product may result in high phthalate exposures (Nilsson et al. 2006).

In their article, Zota et al. (2014) revealed that phthalate exposures in the United States are beginning to change after remaining relatively stable for a decade. The authors reported that urinary metabolite levels of certain phthalates associated with male developmental effects, including di(2-ethylhexyl) phthalate (DEHP) and di-*n*-butylphthalate (DnBP), are decreasing while exposure to other phthalates, diisobutyl phthalate (DiBP) and diisononyl phthalate (DiNP), are increasing. The observed trend is something of a good news–bad news story. The good news is that exposures to DEHP and DBP are declining. The bad news is that exposure to DiBP and DiNP, which are associated with similar health effects, are increasing. However, DiNP is less potent as an antiandrogen than the other phthalates. Furthermore, exposure to phthalate substitutes (e.g., Schütze et al. 2014), as well as unregulated phthalates such as DiBP, are likely to increase. In the future, biomonitoring studies such as the National Health and Nutrition Examination Survey (NHANES) and the National Children’s Study (NCS) may need to shift their focus to substitutes. Like phthalates, many of the phthalate substitutes (including citrates and adipates) are high production volume chemicals, with little adequate toxicological data, and humans are exposed from multiple sources.

Although the authors are members of the Chronic Hazard Advisory Panel on Phthalates and Phthalate Alternatives, U.S. Consumer Product Safety Commission (Division of Toxicology and Risk Assessment), they are writing independently of their panel activities.
